# Functional Analysis of the ComK Protein of *Bacillus coagulans*

**DOI:** 10.1371/journal.pone.0053471

**Published:** 2013-01-03

**Authors:** Ákos T. Kovács, Tom H. Eckhardt, Richard van Kranenburg, Oscar P. Kuipers

**Affiliations:** 1 Molecular Genetics Group, Groningen Biomolecular Sciences and Biotechnology Institute, University of Groningen, Groningen, The Netherlands; 2 Laboratory of Microbiology, Wageningen University, Wageningen, The Netherlands; 3 Kluyver Centre for Genomics of Industrial Fermentation, Groningen, The Netherlands; Loyola University Medical Center, United States of America

## Abstract

The genes for DNA uptake and recombination in Bacilli are commonly regulated by the transcriptional factor ComK. We have identified a ComK homologue in *Bacillus coagulans*, an industrial relevant organism that is recalcitrant for transformation. Introduction of *B. coagulans comK* gene under its own promoter region into *Bacillus subtilis comK* strain results in low transcriptional induction of the late competence gene *comGA*, but lacking bistable expression. The promoter regions of *B. coagulans comK* and the *comGA* genes are recognized in *B. subtilis* and expression from these promoters is activated by *B. subtilis* ComK. Purified ComK protein of *B. coagulans* showed DNA-binding ability in gel retardation assays with *B. subtilis-* and *B. coagulans*-derived probes. These experiments suggest that the function of *B. coagulans* ComK is similar to that of ComK of *B. subtilis*. When its own *comK* is overexpressed in *B. coagulans* the *comGA* gene expression increases 40-fold, while the expression of another late competence gene, *comC* is not elevated and no reproducible DNA-uptake could be observed under these conditions. Our results demonstrate that *B. coagulans* ComK can recognize several *B.* *subtilis comK*-responsive elements, and *vice versa*, but indicate that the activation of the transcription of complete sets of genes coding for a putative DNA uptake apparatus in *B. coagulans* might differ from that of *B. subtilis*.

## Introduction

The ability to take-up DNA from the environment is widely spread among eubacteria, including Gram-positive and Gram-negative species [1]. It allows the exchange of genetic material, possibly contributing to the survival of bacteria under harsh growth conditions [2]–[4]. Cells that activate the expression of genes coding for a DNA uptake and recombination apparatus can benefit from foreign DNA after it recombines into the genome. Due to the need for homologous sequences for recombination it is proposed that DNA is utilized more efficiently from closely related species [5]. The induction of the competence genes has been studied in various bacteria [2], [6]. In Gram-positives, a global transcription factor or sigma factor coordinate the expression of genes required for efficient DNA uptake and recombination, the so-called late competence genes. In Streptococci, the conserved ComX sigma factor activates the late competence genes [5], while the global transcription factor ComK has been identified in various Bacilli to activate gene expression of genes related to DNA uptake [7]. As the induction of functional DNA uptake can be a useful tool for molecular biotechnological applications, numerous studies aim to better characterize the regulators involved in competence and try to achieve highly transformable strains [8]–[14].

The genes coding for DNA uptake and recombination are conserved among Bacilli [7]. The functional uptake of exogenously provided genomic DNA has been shown in various strains of *B. subtilis* [8], [12], [15], and also in other *Bacilli*, like *B. licheniformis* [10], [16], *B. amyloliquefaciens* [17], and *B. cereus* [11]. The regulation and function of late competence genes have been mainly studied in *B. subtilis* [18]. The 7 genes containing *comG* operon encodes a type IV pilus that facilitates DNA to pass the cell wall and reach the cell membrane [19]. The maturation of the pilin like proteins is facilitated by the ComC prepilin protease [20]. DNA is bound and transported across the membrane in a single stranded form by the ComEA protein and ComEC permease, respectively, with the aid of ComFA and NucA proteins [18]. The single-stranded DNA is then integrated via recombination by a protein complex containing among others RecA, SsbB, DprA and YjbF [21].

The late competence genes are scattered around the *B. subtilis* chromosome. To coordinate the expression of these genes and operons, *B subtilis* utilizes the global transcription factor ComK. If the protein level of ComK increases in the cells, ComK directly or indirectly activates more than 100 genes [6], [22]–[24]. ComK binds to the so-called K-boxes, that contains two AT-boxes (AAAA-N_5_-TTTT) separated by a spacer of a discrete number of helical turns [25]–[27]. To ensure that competence develops only under particular conditions, the expression of the *comK* gene and the protein level of ComK are tightly regulated. Transcription of *comK* is repressed by AbrB, CodY, and Rok and activated by the DegU protein [6], while the ComK protein is trapped by the adaptor protein MecA, and targeted to proteolysis by ClpCP [28]. At high cell densities, the expression of the *comS* gene, embedded in the *srfA* operon, is activated in a quorum sensing dependent manner [29]. ComS protein hijacks the MecA protein and prevents ComK degradation [28]. The increase of ComK amounts in the cells leads to a positive feedback loop and the protein level further increases. However, this enhanced level of ComK is only developed in a subpopulation of cells [30], [31]. The occurrence of two subpopulations of cells with a distinct expression state is called bistability [32] and has not been only described for competence, but also for other phenotypes of *B. subtilis*, like sporulation, motility, biofilm formation, and protease production [33]–[37].

In this study we characterized the function of the *Bacillus coagulans* ComK homologue in *B.* *coagulans* and in *B. subtilis*. *B. coagulans* is a spore forming, microaerophilic, lactic acid producing species of the *Bacillus* genus. It is frequently isolated as food spoilage organism [38], while its propitious features are used in probiotics [39]. It can be applied as a lactic acid production organism in biotechnological procedures and various molecular tools have been developed recently [40]–[42]. The genome sequences of several strains have been determined that facilitate genomics studies in this group of organisms [43]–[45]. As there is no study published on DNA uptake in *B. coagulans*, our aim was to better characterize the ComK homologue from *B. coagulans* DSM 1. First, we assayed the *B. coagulans comK* gene (denoted as *comK_Bco_*) and promoter regions of *comK_Bco_* and *comG_Bco_* in the heterologous host, *B. subtilis*. *In vitro* studies further supported the conserved role of ComK*_Bco_* as a DNA binding protein. Finally, we assayed the effect of *comK_Bco_* overexpression in *B. coagulans*.

## Results

### Identification of *comK* Homologue in *B. coagulans*

**Figure 1:**
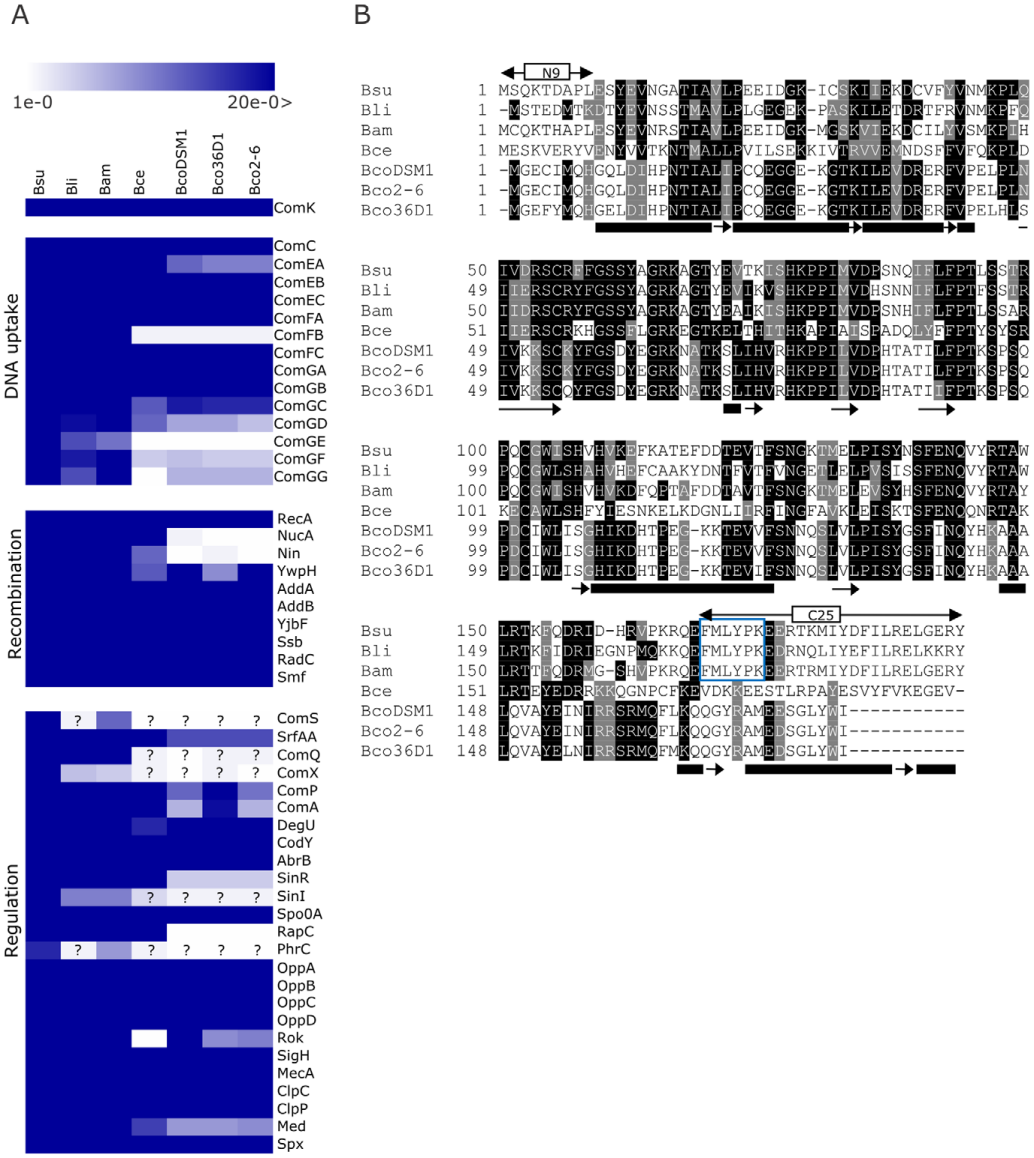
Survey on the presence of competence genes and the alignment of ComK protein sequences from various *Bacillus* strains. (A) Results of BLAST searches were visualized with Genesis 1.6 software: white is absent (with E-value of E–0), dark blue is present (E-value<E–20). BLAST analysis was performed with *B. subtilis* protein sequences against translated protein database of a given genome. Protein names are indicated on the right. Bsu, *B. subtilis*; *Bli*, *B. licheniformis*, *Bam*, *B. amyloliquefaciens*, Bce, *B. cereus* Bco, *B. coagulans*. Question marks denote small ORFs where identification is uncertain using the available bioinformatic tools that can miss homologues. (B) Multiple alignment of ComK homologues. Black background represents conserved amino acids and grey background represents similar amino acids. Alignment was performed using ClustalW [59], and presented using Boxshade 3.21 program. The N- and C-terminal deletions analyzed by Susanna et al [47] are marked (ΔN9 and ΔC25, respectively). Boxed amino acid residues indicate the residues involved in interaction with MecA [60]. Alpha-helices and beta-sheets of *B. subtilis* ComK protein are indicated with rectangles and arrows under the alignment, respectively.

Genomic inspection of various *B. coagulans* strains, including DSM 1 (unpublished data), 36D1 and 2–6 showed that several genes and operons can be identified with high sequence similarity to *Bacillus* genes that code for the late competence genes in *B.* *subtilis* and their homologues in other *Bacillus* species (Fig. 1A). BLAST analyses revealed the presence of many orthologous genes putatively involved in DNA uptake and recombination, which we visualized with Genesis software. As in the case of many *Bacillus* species [7], the *comFB* gene is missing in the *comF* operon. While the putative ComGEFG proteins lack high similarity to the corresponding proteins of *B. subtilis*, the number of genes in the *comG* operon is conserved and the coded proteins show higher similarity to the corresponding proteins of *B. cereus*, where functional DNA uptake has been shown [11]. Interestingly, the genomes of all *B. coagulans* species also lack the *nucA*-*nin* operon that is required for the DNA cleavage during transformation in *B.* *subtilis* [46]. The absence of these genes reduces transformation efficiency to 8–15% of the wild type in *B. subtilis*. Still, the genomic analysis of competence genes shows that homologues of the majority of genes coding for the *B. subtilis* DNA-uptake and recombination machinery are present and conserved in all *B.* *coagulans* strains.

Homologues of the *comK* gene are present in all *B. coagulans* species. Although the *comK* homologue is not annotated in the complete genome of *B. coagulans* 2–6, a gene that codes for a putative ComK homologue can be identified between nucleotides 860419 and 860961 of the *B. coagulans* 2–6 chromosome (NCBI reference sequence NC_015634.1). The *B. coagulans* ComK homologues are somewhat shorter than the ComK protein of *B.* *subtilis* (13 aminoacids shorter compared to ComK of *B. subtilis*), but most regions are conserved (Fig. 1B). The C-terminal region of *B. coagulans* ComK proteins is truncated by 11 amino acids. Previous studies have shown that a 25–35 amino acid C-terminal truncation is incapable of transcriptional induction of *comG* operon [47]. The ComK proteins of *B. coagulans* strains have half of this C-terminal part. Interestingly, as shown in many *Bacillus* species, the region recognized by the adaptor protein MecA is not conserved in any of the *B. coagulans* species suggesting that the interaction site is different or that the ComK level is not controlled by a MecA homologue in *B. coagulans* species, while putative MecA homologues are present in all *B. coagulans* strains (Fig. 1A).

Examination of the presence of the early regulatory competence genes suggest that pleiotropic regulators (DegU, CodY, AbrB, and Spo0A) that directly or indirectly control *comK* transcription in *B.* *subtilis* are present in *B. coagulans*, while SinR and the Rap-Phr signaling systems seem to be less conserved or absent in *B. coagulans* (Fig. 1A). Interestingly, *rok* can be identified in *B. coagulans*, while it was previously described to be present only in the *B. subtilis/amyloliquefaciens/pumilus/licheniformis* group [7].

### Introduction of *comK_Bco_* into *B. subtilis* Results in Activation of Gene Expression from P*comGA_Bsu_*

**Figure 2:**
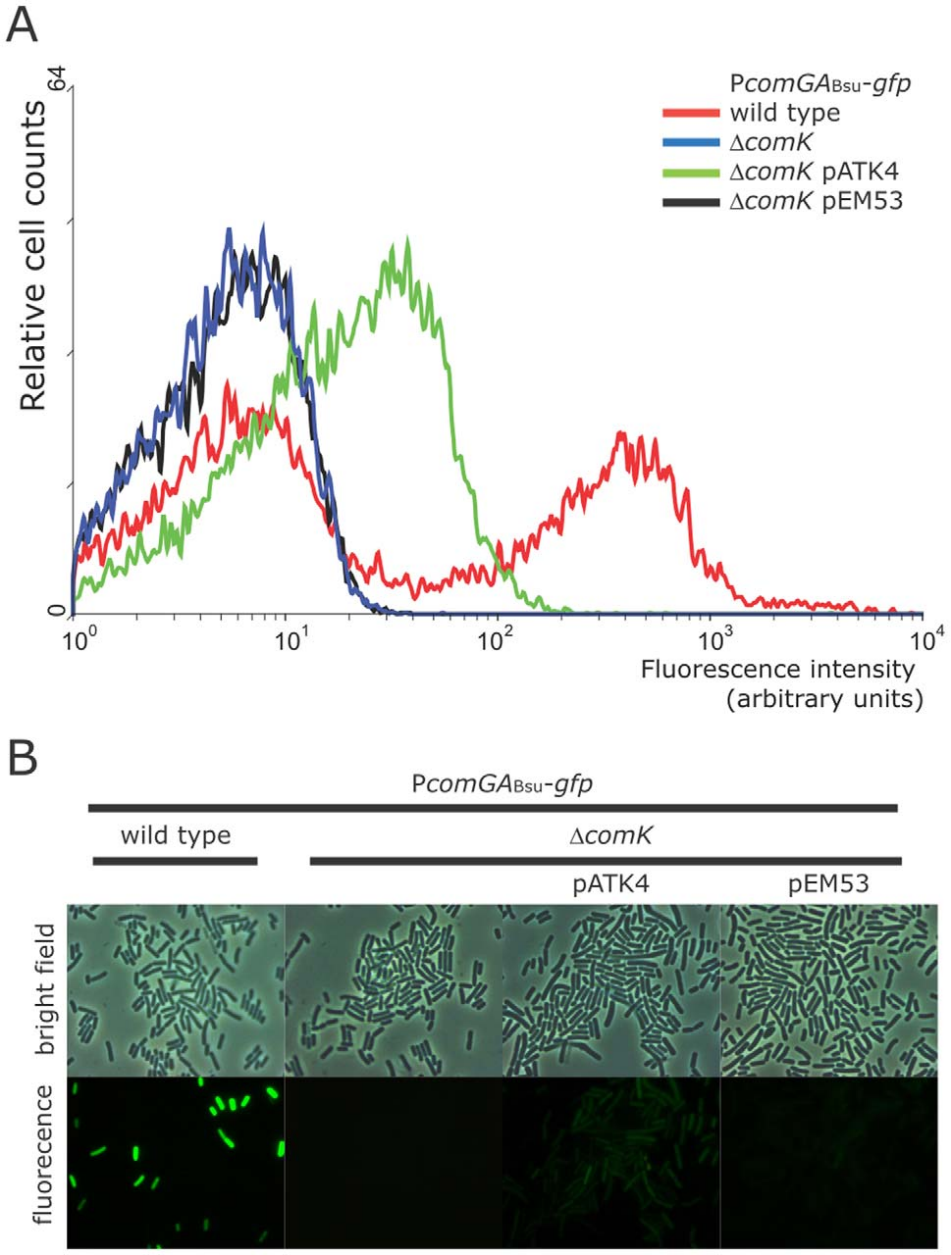
Single cell analysis of P*comGA_Bsu_*-*gfp* in the presence of *comK_Bco_* in *B. subtilis*. Samples were taken 2 hours after the transition point between the exponential and stationary growth phase. (A) Flow cytometric analyses of *comGA_Bsu_* expression in wild type (red line), Δ*comK* mutant (blue line), Δ*comK* strain with the *comK_Bco_* containing plasmid pATK4 (green line), and Δ*comK* strain with the empty plasmid (black line). The relative numbers of cells are indicated on the *y* axis, and their relative fluorescence levels are indicated on the *x* axis on a logarithmic scale. For each experiment at least 20,000 cells were analyzed. The graph is the representative of at least three independent experiments. (B) Light-microscopic phase-contrast picture (top row) and fluorescence image (bottom row) of cells. Strains used from left to right are wild type, Δ*comK* mutant, Δ*comK* with pATK4, and Δ*comK* with pEM53, respectively.

On the basis of its protein sequence analysis, the *comK_Bco_* gene of *B. coagulans* DSM1 appears to code for another member of the ComK family. Therefore, we wanted to test if the product of the *comK* gene can activate transcription. For this we first introduced the *comK_Bco_* gene (cloned in pATK4) into *B. subtilis* harboring a P*comGA_Bsu_*-*gfp* reporter that enables us to monitor the activation of gene expression. The expression of *comK_Bco_* is driven by its own promoter region. Subsequently, we deleted the endogenous *comK_Bsu_* gene in this strain so we can solely monitor the effect of *comK_Bco_*. In this synthetic background, the reporter activity observed depends on the presence of ComK*_Bco_*. As depicted in Fig. 2, we observed reporter activity from the *comGA_Bsu_* promoter when the *comK_Bco_* gene was introduced, but not when the empty plasmid was present. The gene expression was detected using both flow cytometry (Fig. 2A) and fluorescence microscopy (Fig. 2B). The expression from P*comGA_Bsu_* was low compared to the strain in which wild type *comK_Bsu_* was present, and expression of *comGA_Bsu_* was not bistable in contrast to the expression observed in the wild type *B. subtilis* strain. However, the lack of bistable gene expression of the reporter gene could also originate from a low expression level from the *comK_Bco_* promoter in *B. subtilis*. These experiments suggest that *comK_Bco_* is able to affect gene expression in *Bacilli*. Introduction of *comK_Bco_* into *B. subtilis* resulted in low *comGA_Bsu_* expression, which suggests the lack of complete functional complementation of *comK_Bsu_* deletion under the tested conditions. Accordingly, no natural transformation was observed in the complemented *B. subtilis* strain (data not shown).

### ComK_Bsu_ Activates Transcription from the Promoter Regions of *comK_Bco_* and *comGA_Bco_*

**Figure 3:**
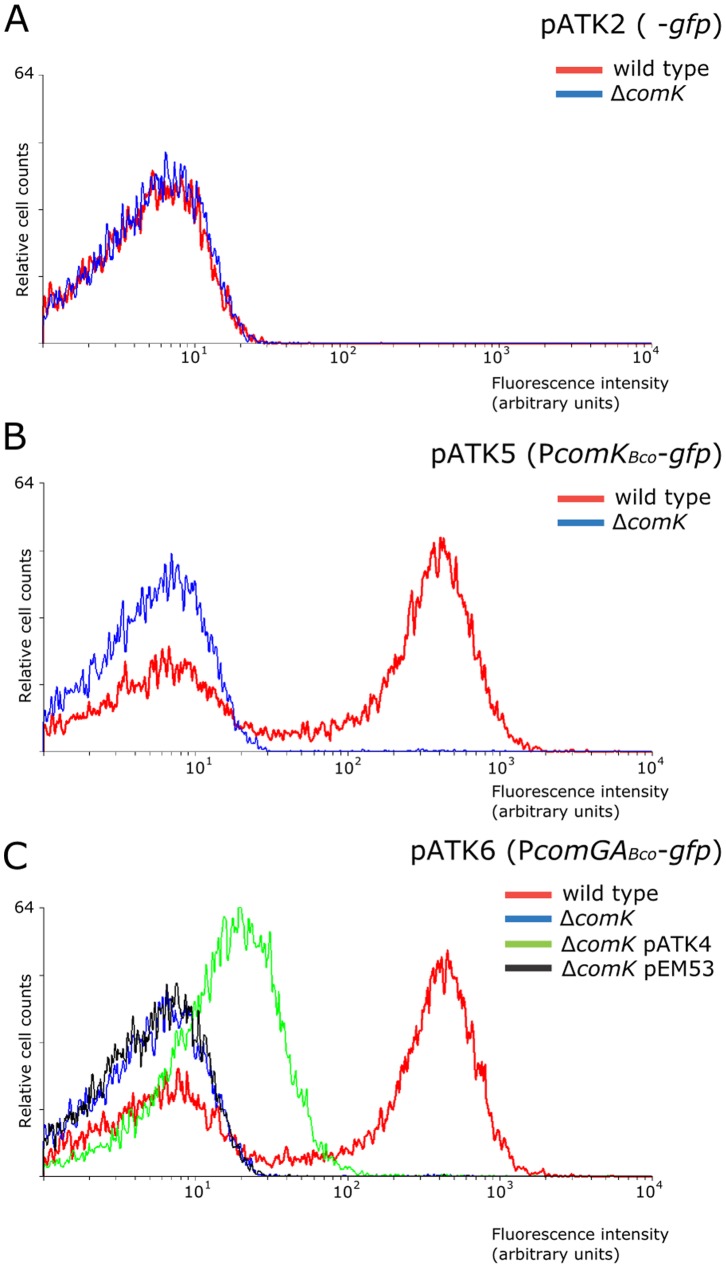
Expression from the *comK_Bco_* and *comGA_Bco_* promoters in *B. subtilis*. Single cell analysis of *B. subtilis* strains containing plasmids with promoter-less *gfp* (A), with P*comK_Bco_*-*gfp* fusion (B), and the P*comGA_Bco_*-*gfp* reporter (C). Samples were taken at the indicated time points given in hours relative to the transition point between the exponential and stationary growth phase (T0). The single cell expression pattern in the wild type strain is indicated with light grey, the Δ*comK* mutant is designated with dark grey, and the Δ*comK* strain with the *comK_Bco_* containing plasmid pATK4 is shown in white. The relative numbers of cells are indicated on the *y* axis, and their relative fluorescence levels are indicated on the *x* axis on a logarithmic scale. For each experiment at least 20,000 cells were analyzed. The graph is the representative of at least three independent experiments.

ComK*_Bco_* can activate gene expression in the heterologous host *B. subtilis*. The transcription activation by ComK proteins depends on the promoter sequences they bind and their relative amount, and they either activate gene expression (e.g. *comGA_Bsu_* promoter [26]) or relieve transcription repression (e.g. *comK_Bsu_* promoter [48]). To test whether the elements of the *comGA_Bco_* and *comK_Bco_* promoters are functionally conserved, we assayed the effect of the ComK*_Bsu_* protein on these promoter fragments in the heterologous host, *B. subtilis*. For this we introduced the promoter-*gfp* constructs pATK5 and pATK6 into *B. subtilis* and subsequently also assayed the effect of the *comK_Bsu_* mutation on the expression from these promoters. Expression of a reporter gene from both the *comK_Bco_* and *comGA_Bco_* promoters was observed in *B. subtilis* (Fig. 3). This expression was dependent on the presence of the ComK*_Bsu_* protein. The activation of gene expression from the introduced promoters showed a bimodal expression pattern that could originate from the bimodal level of ComK*_Bsu_* protein in *B. subtilis* or due to use of plasmid based system to monitor gene expression. However, we can conclude that the *comGA_Bco_* and *comK_Bco_* promoters are recognized in *B. subtilis* in a *comK_Bsu_*-dependent manner. Introduction of *comK_Bco_* (pATK4) into the Δ*comK_Bsu_* strain containing the pATK6 plasmid showed that ComK*_Bco_* can activate gene expression from the promoter of *comGA_Bco_* (Fig. 3C, pATK6, Δ*comK_Bsu_* with pATK4) similarly to that observed for the *comGA_Bsu_* promoter (Fig. 2A, P*comGA_Bsu_*-gfp, Δ*comK_Bsu_* with pATK4).

### ComK*_Bco_* is a DNA Binding Protein

**Figure 4:**
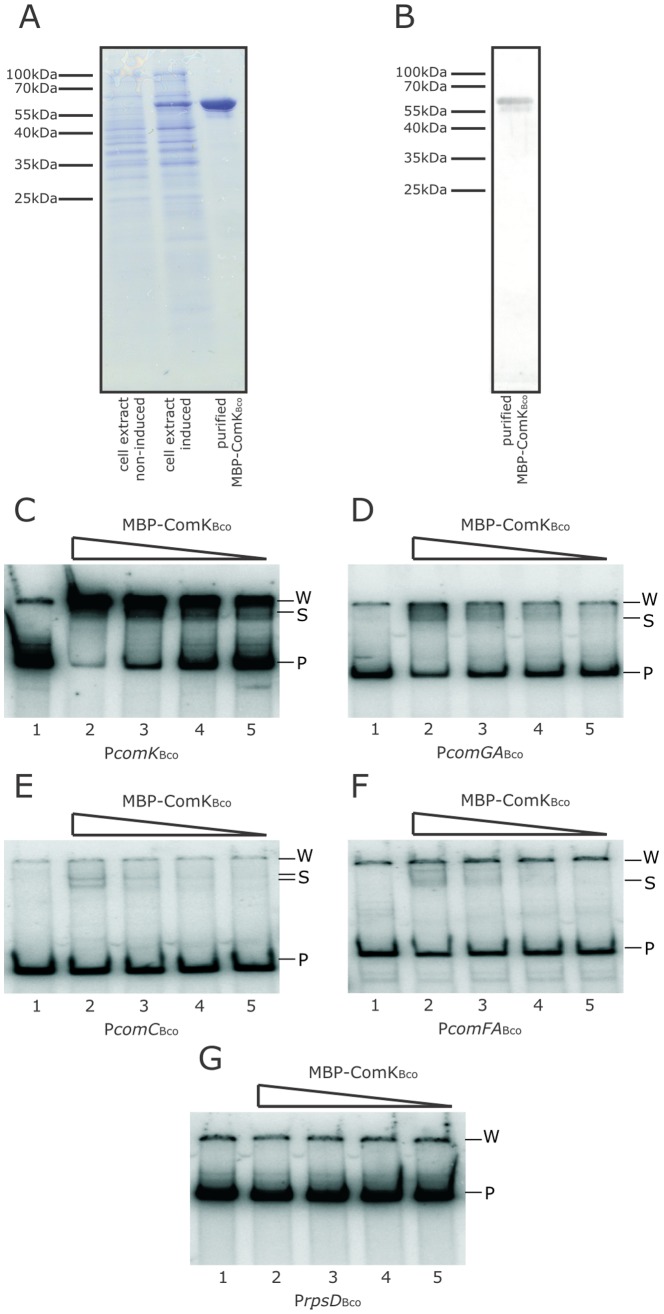
Purification of ComK*_Bco_* protein and its DNA binding ability. (A) SDS-PAGE analysis of overexpression and purification of ComK*_Bco_* protein from *E. coli*. Non-induced and 0.1 mmol l^−1^ IPTG induced cell extracts are loaded on the first and second lanes, respectively, while purified MBP-ComK_Bco_ is run in the third lane. (B) Immunoblot analysis of the purified MBP-ComK*_Bco_* protein using α-ComK*_Bsu_* antibodies. Marker sizes are indicated on the left of the blot. (C–G) Gel retardation assay with the purified MBP-ComK*_Bco_* protein on the *comK_Bco_* (C), *comGA_Bco_* (D), *comC_Bco_* (E), and *comFA_Bco_* (F) promoter fragments. Lane 1 contains no protein, lanes 2–5 contain 2.2 µmol l^−1^ to 275 nmol l^−1^ purified MBP-ComK_Bco_ at 2 fold dilutions, respectively. The promoter fragment of *rpsD_Bco_* is used as negative control with no apparent binding by MBP-ComK*_Bco_* (G). Free probes (P), shifted bands (S), and signal specific to the wells of the gel (W) are indicated.

Experiments presented above show that ComK_Bco_ affects expression from the *comGA_Bsu_* promoter and that the *comGA_Bco_* and *comK_Bco_* promoters are also recognized by ComK*_Bsu_*. To test this in more details, we examined the *in vitro* DNA binding ability of ComK*_Bco_*. We overexpressed a *malE*-*comK_Bco_* fusion construct in *Escherichia coli* and purified the ComK*_Bco_* protein with the aid of the maltose binding protein (MBP) tag (Fig. 4A). MBP fusion tag is generally used to purify DNA binding proteins and assay the *in vitro* DNA binding ability of target proteins. The MBP tag did not alter the binding ability of ComK*_Bsu_* protein in previous studies [25]. As a control, we also obtained the MBP-ComK*_Bsu_* protein using the same purification procedure. The MBP-ComK*_Bco_* protein was overexpressed in *Escherichia coli* and purified as described in the Methods section (Fig. 4A). The integrity of the purified MBP-ComK*_Bco_* protein was also verified using antibodies developed against the ComK*_Bsu_* protein (Fig. 4B). A smaller protein band was copurified and recognized by the ComK*_Bsu_*-antibody. The purified MBP-ComK*_Bco_* clearly bound to the DNA fragment containing the *comK_Bco_* and *comGA_Bco_* promoter regions in gel retardation assay (Fig. 4C and D). We also observed a weaker DNA binding of MBP-ComK*_Bco_* to the *comC_Bco_* and *comFA_Bco_* promoters (Fig. 4E and F). The MBP-ComK*_Bco_* showed no binding towards the *B. coagulans rpsD* promoter fragment that is used as a non-specific control in our experiments (Fig. 4G).

**Figure 5:**
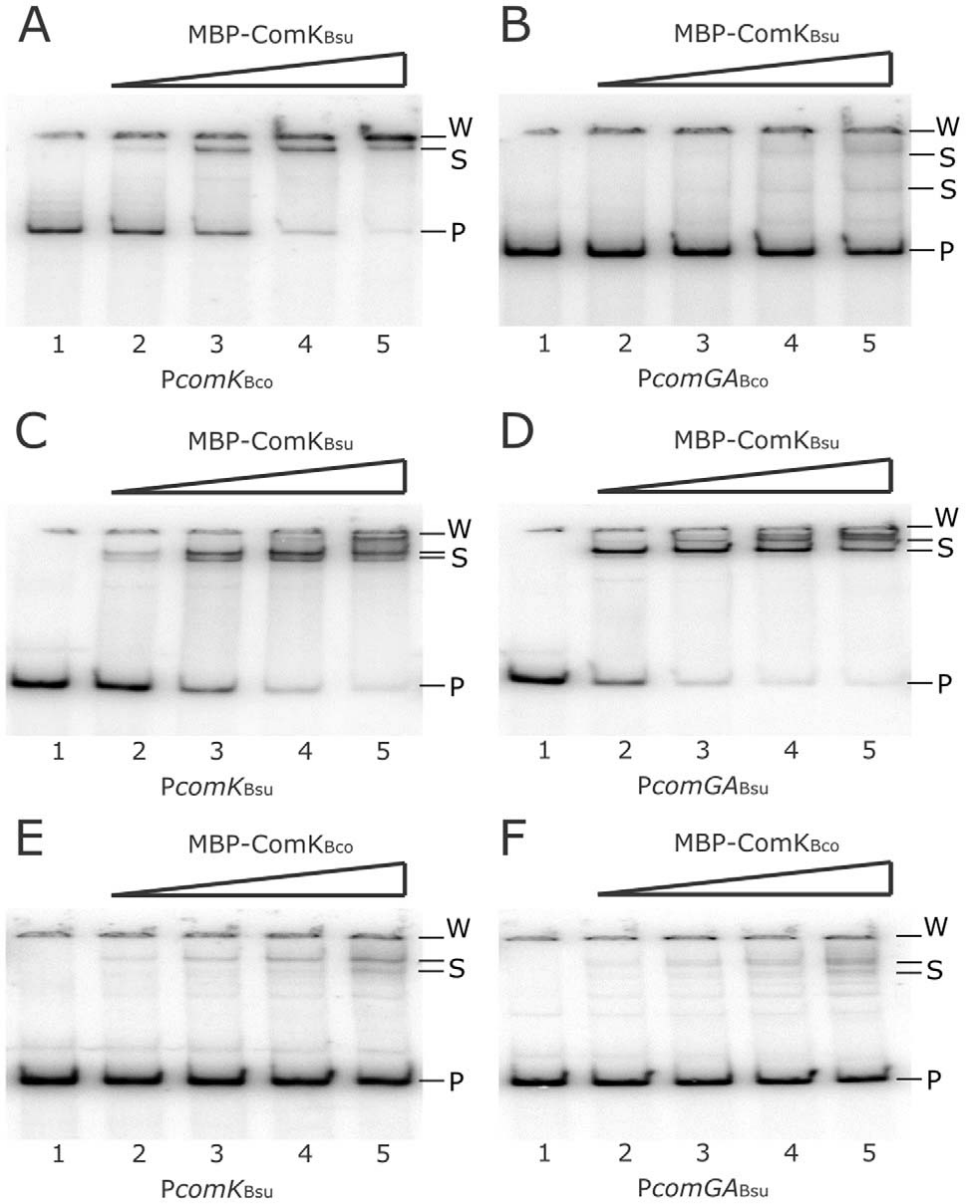
Gel retardation assay with ComK*_Bsu_* and ComK*_Bco_*. The binding of MBP-ComK*_Bsu_* (A–D) and MBP-ComK*_Bco_* (E–F) was assayed at a doubling concentration of the proteins from 125 nmol l^−1^ to 1 µmol l^−1^ (lanes 2 to 5, respectively). Lane 1 of each picture lacks any added protein. DNA binding was detected on promoters of *comK_Bco_* (A), *comGA_Bco_* (B), *comK_Bsu_* (C and E), and *comGA_Bsu_* (D and F) genes. Free probes (P), shifted bands (S), and signal specific to the wells of the gel (W) are indicated.

Experiments performed in *B. subtilis* suggest that the ComK proteins can activate gene expression on the heterologous *comGA* promoters (see above). To examine if this effect of ComK proteins is achieved by direct binding and transcription activation, we examined the *in vitro* binding of various ComK proteins on the *B.* *coagulans* and *B. subtilis* promoters of the *comGA* and *comK* genes. Results depicted in Fig. 5 show that the ComK proteins bind to the heterologous promoters, although the affinity of the ComK proteins was different in the case of different promoters. As our gel retardation experiments were not controlled in competition experiments with a cold probe, we can only judge the presence of DNA binding, but no indisputable conclusion can be drawn on the affinity differences. However, the binding of ComK proteins of *B. coagulans* and *B. subtilis* to the heterologous promoter fragments is in agreement with the *in vivo* experiments done in *B. subtilis*. Taken together the *in vivo* and *in vitro* experiments all suggest that *B. coagulans* possesses a functional ComK homologue that is presumably able to activate the transcription of several late competence genes in *B. coagulans* (see also below).

ComK*_Bsu_* activates transcription by binding K-boxes that are composed of two AT-boxes with a consensus sequence AAAA-N_5_-TTTT. The boxes are separated of a discrete number of helical turns (8-, 18- or 31-bp between the two AT-boxes), which places them on the same side of the DNA-helix [25]–[27]. The analysis of the promoter region of putative competence related genes in *B.* *coagulans* showed the presence of several AT-boxes (allowing maximum 3 mismatches to the consensus AT-box), however, K-boxes could be only found in the promoter regions of *comK_Bco_* and *comC_Bco_* (Figure S1). Interestingly, the promoter regions of *comC_Bco_*, *comEA_Bco_* and *comFA_Bco_* contain an overrepresented GCC-N_8_-TGC motif (identified 1, 2, and 3 times, respectively). This motif is not found within the promoter regions of the *comGA_Bco_* and *comK_Bco_* genes. However, due to the low number of analyzed promoters, we cannot conclude whether the K-boxes or this latter overrepresented motif are functional in *B. coagulans* and their role requires additional functional characterization.

### Overexpression of *comK_Bco_* in *B. coagulans* Results in Elevated *comGA_Bco_* Expression

In our final experiments, we assayed the effect of *comK_Bco_* overexpression in *B. coagulans* DSM 1. For this, we cloned the *comK_Bco_* gene under control of the IPTG (isopropyl-β-d-thiogalactopyranoside) inducible *spac* promoter, resulting in plasmid pATK10. We introduced this construct into *B. coagulans* DSM 1 by electroporation and assayed whether the level of ComK protein is enhanced in *B. coagulans* containing pATK10 upon induction. An increased level of ComK protein was detected in Western blot analysis using antibodies against ComK_Bsu_ (Figure S2). Next, we monitored the expression of late competence genes using quantitative RT-PCR. As expected, the expression level of *comGA_Bco_* gene was increased (ratio of 30.5±3.7) in the strain where *comK_Bco_* expression was induced with 1 mM IPTG compared to the wild type strain that lacks the plasmid. However, the expression level of another late competence gene (i.e. *comC_Bco_*) showed no significant change (ratio of 0.85±0.3) in the *comK_Bco_*-overexpression strain compared to the plasmid-free strain under this given condition. Other late competence genes (*comEA-C_Bco_* and *comFAC_Bco_*) also lacked the increased expression in the *comK_Bco_* overexpression strain (data not shown). Overexpression of the *comK_Bsu_* gene using the previously published pNW*comK_Bsu_* plasmid [11] resulted in slightly increased *comGA_Bco_* expression (ratio of 3.2±1.2) and unaltered *comC_Bco_* transcription (ratio of 1.3±0.5) compared to the plasmid free wild type strain. These experiments demonstrate that ComK*_Bco_* can activate gene expression in *B.* *coagulans* in line with previous observations presented above.

To test if the increased expression of *comGA_Bco_* by *comK_Bco_* overexpression is sufficient to observe a functional DNA uptake in *B. coagulans*, we tested the uptake of genomic DNA (e.g. chromosomal DNA of DSM1 Δ*sigF*::Cm^r^ described in [40]) or plasmid DNA (e.g. pNW33N). The expression of *comK_Bco_* was induced at mid-exponential phase and DNA was supplied at different time points (1–4 hours) after induction. Cells were plated on medium containing chloramphenicol. We could not observe reproducible DNA uptake under the above presented *comK_Bco_* overexpressing conditions in *B. coagulans*, suggesting the lack of a fully functional DNA uptake machinery under these specific conditions. Similarly, DNA uptake was not detected in *B. coagulans* when *comK_Bsu_* was overexpressed, in contrast to the experiments with *B. cereus* [11]. Since an increased level of ComK*_Bco_* is detected by Western blot analysis when *comK_Bco_* is overexpressed in *B.* *coagulans* (Figure S2) and the *comGA_Bco_* gene expression was induced roughly 30 times, it may be that the resulting level of ComK*_Bco_* is not high enough to activate the whole DNA-uptake and recombination apparatus.

## Discussion

Genetic engineering of microorganisms allows to improve them or introduce alternative biochemical reactions and thereby to develop improved or novel strains or products. However, genetic engineering can be time consuming for recalcitrant bacteria. The use of competence for DNA uptake and recombination improves the engineering process by allowing or enhancing genetic accessibility. Competence has been described for many laboratory type strains of Bacilli [15]–[17] [12]. The genes coding for functional DNA uptake and recombination are widely conserved in Bacilli suggesting that natural competence exists in more species than described before [7]. However, highly efficient DNA uptake is not identified under laboratory conditions in many species. Different strains of the same species might also differ in their degrees of competence. Natural isolates of *B. subtilis* show a low DNA uptake efficiency that can be improved by induction of the late competence genes through overexpression of the *comK* gene [12].

In this study, we present the genomic conservation of genes coding for putative homologues for DNA uptake and the recombination apparatus in *B. coagulans*. Further characterization of the *comK* homologue in *B. coagulans* DSM 1 indicates that *comK_Bco_* codes for a DNA binding transcriptional activator. Introduction of *comK_Bco_* into a synthetic *B. subtilis* background that lacks its own *comK_Bsu_* gene results in gene expression activation from the promoter regions of *comG* operons of *B. subtilis* and *B. coagulans*. These experiments clearly suggest a conserved role of ComK homologues in Bacilli, although the set of target genes might vary. This is also supported by the induction of functional DNA uptake in *B. cereus* by the ComK*_Bsu_* protein [11]. However, overexpression of either or both *comK* genes of *B. cereus* into *B. subtilis* does not result in a similar induction of *comG* expression (unpublished observation, AM Mironczuk and ÁT Kovács). Previous studies on the binding site of ComK*_Bsu_* described K-boxes, where the distance between the two AT-boxes is important for its function [25], [26]. While AT-boxes can be identified in several promoter regions of late competence genes in various Bacilli, properly spaced K-boxes are found only in the promoter regions of *comK_Bco_* and *comC_Bco_* genes. In contrast with these *in silico* observations, purified ComK*_Bsu_* protein binds *in vitro* to the promoter regions of late competence genes of *B. coagulans* (Fig. 5) and *B. cereus* [11] and overexpression of *comK_Bsu_* results in enhanced *comG* expression *in vivo* (RT-qPCR results and [11]). This suggests that the recognition and transcriptional activation by ComK proteins might not be so stringent in the heterologous hosts. Alternatively, ComK proteins of *B. coagulans* and *B. cereus* could act on deviating binding sites or their effect is indirect on the *comG* promoter.

Overexpression of various *comK* genes in different Bacilli results in increased transcription from the *comG* promoter. In the present and previous studies, we used the fusion between the *comG* promoter and the reporter gene *gfp*, for general use of *comG_Bsu_* expression as a reporter of activation of competence in *B. subtilis* [30], [31], [49]. However, microarray analysis and RT-qPCR experiments in *B. cereus* showed that while expression of *comK* genes increases *comG* transcription, the transcript levels of other late competence genes are not induced equally [11]. In *B. coagulans*, when the ComK*_Bco_* protein level is increased to a certain level that results in roughly 30 times induction of *comGA_Bco_*, the expression of *comC_Bco_* is not changed. We can hypothesize that the produced ComK protein level is not high enough to activate gene expression from these promoters or one or more additional regulatory mechanisms act on the late competence genes. *In vitro* transcription assays using these promoter regions and purified ComK protein could show us whether this is the case.

While overexpression of *comK* genes in *B. coagulans* results in increased *comG* expression similar to the experiments in *B. cereus* [11], we did not detect functional uptake of DNA under these conditions. Our survey on the presence of late competence genes in *B. coagulans* also points to the absence of genes that are required for high efficiency DNA uptake in *B. subtilis* (e.g. *nucA-nin* genes). However, our study clearly shows that ComK*_Bco_* is a DNA-binding protein that is capable of activating gene expression. Therefore, it presents an important element of future research for better understanding of late competence gene induction in *B. coagulans*.

## Methods

### Bacterial Strains, Growth Conditions and Transformation

The strains and plasmids used in this study are listed in Table 1. *B. coagulans* strains were grown in BC medium at 50°C, 120 rpm [40]. BC medium contains per liter: 10 g yeast-extract (Difco), 2 g (NH_4_)_2_HPO_4_, 3.5 g (NH_4_)_2_SO_4_, 10 g Bis-Tris (bis[2-hydroxymethyl]iminotris[hydroxymethyl]-methane), 5 mg MgCl_2_ · 6 H_2_O, 3 mg CaCl_2_ · 2 H_2_O, 1 ml of filter sterilized trace elements (containing per liter 0.05 g ZnCl_2_, 0.03 g MnCl_2_ · 4 H_2_O, 0.3 g H_3_BO_3_, 0.2 g CoCl_2_ · 6 H_2_O, 0.01 g CuCl_2_ · 2 H_2_O, 0.02 g NiSO_4_ · 6 H_2_O, and 0.03 g Na_2_MoO_4_ · 2 H_2_O), pH 6.7. *B. subtilis* strains were grown in minimal medium [15]. For cloning, *Escherichia coli* DH5α and *Lactococcus lactis* MG1363 were grown in TY and GM17 (37.5 g M17 broth (Difco) per liter supplemented with 0.5% glucose) medium, respectively, grown at 30°C or 37°C. Antibiotics were used at a concentration of 5 µg ml^−1^ for chloramphenicol, 6 µg ml^−1^ for tetracycline, and 100 µg ml^−1^ for ampicillin. Transformation of *L. lactis* and *B.* *coagulans* was performed by electroporation as previously described [40], [50]. Transformation of *E. coli* was performed by heat-shock [51]. DNA was introduced into *B. subtilis* strains using natural competence [52].

**Table 1:** Strains, plasmids used in this study.

	Properties	Reference
**Strain**		
*B. coagulans* DSM 1	wild type strain	DSMZ collection
*B. subtilis* 168	wild type strain	laboratory strain
*B. subtilis* Δ*comK*	*comK*::Km^r^ mutant	[30]
*B. subtilis* P*comG*-*gfp*	P*comG-gfp* fusion in *B. subtilis* 168 strain (Cm^r^)	[30]
*L. lactis* MG1363	lac^−^ prt^−^; plasmid-free derivative of NCDO712	[61]
*E. coli* DH5α	*endA1 hsdR17 supE44 thi-1 λ* ^−^ *recA1 gyrA96 relA1 ΔlacU169* (φ80d*lacZ*ΔM15)	Bethesda Research Laboratories
**Plasmids**		
pNW33N	4.2 kb, Cm^r^, *Geobacillus*-*E. coli* shuttle vector	Bacillus Genetic Stock Centre
pEM53	5.6 kb, Tc^r^, pNZ124-based cloning vector	[40]
pDG148	8.3 kb, Amp^r^, Km^r^,P*spac*, *lacI* integration vector	[62]
pMALc2X	6.6 kb, Amp^r^, overexpression vector for MalE fusion	New England Biolabs
pSG1151	4.6 kb, Amp^r^, Cm^r^, *gfpmut*1 harboring plasmid	[63]
pATK2	5.0 kb, Cm^r^, *gfpmut*1 cloned into pNW33N	This study
pATK4	4.9 kb, Tc^r^, *comK_Bco_* gene and promoter region in pEM53	This study
pATK8	4.5 kb, Tc^r^, P*spac*-*comK_Bco_* in pEM53	This study
pATK10	5.8 kb, Tc^r^, P*spac*-*comK_Bco_* overexpression construct and *lacI* in pEM53	This study
pATK5	5.6 kb, Cm^r^, P*comGA_Bco_*-*gfp* fusion	This study
pATK6	5.5 kb, Cm^r^, P*comK_Bco_*-*gfp* fusion	This study
pMAL*comK_Bco_*	7.3 kb, Amp^r^, MAL-ComK overproduction vector	This study

Cm^R^, chloramphenicol resistant;

Tc^R^, tetracycline resistant,

Km^R^, kanamycine resistant,

Amp^R^, ampicillin resistant.

### Cloning of *comK*_Bco_ Gene

To facilitate the purification of MBP-ComK*_Bco_*, the *comK_Bco_* gene was PCR amplified from the genome of *B. coagulans* DSM 1 using oligos oATK26 and oATK14 (for the sequences of oligos, see Table 2) containing BamHI and SalI sites, respectively. The construct pMALcomK was created by ligating the BamHI and SalI digested PCR into the corresponding site of the pMAL-c2 (New England Biolabs). The *comK*_Bco_ gene harboring its own promoter was PCR amplified with oligos oATK1 and oATK2, and cloned into the ScaI site of pEM53 vector, resulting pATK4. The cloned fragment contains the *comK_Bco_* gene and the 732 bp upstream region. Vector pEM53 is derived from pNZ124 by replacing the chloramphenicol resistance gene *cat* by the tetracycline resistance gene *tetK* amplified from pGhost8::IS*S1* [40]. To overexpress *comK_Bco_* in *B. coagulans*, the *comK_Bco_* gene was cloned after the Pspac promoter. For this, the *comK_Bco_* containing PCR fragment was obtained with oATK13 and oATK14 oligonucleotides (Table 2), digested with HindIII-SalI enzymes, and ligated together with the Pspac containing EcoRI-HindIII fragment from pDG148 and the EcoRI-XhoI digested vector, resulting in pATK8. Subsequently, the *lacI* gene was introduced from pDG148 (1294 bp BamHI-SwaI fragment) into the BamHI-ScaI digested pATK8 vector, resulting pATK10. The resulting vectors were validated using restriction analysis and inserts were verified by sequencing.

**Table 2:** Oligonucleotides used in this study.

Oligo name	target	Sequence (5′ - 3′)	Restriction site
oATK1	*comK_Bco_*	GGACCGTTACGCCGTAGAGA	
oATK2	*comK_Bco_*	GGACTTGCAGTTCGCAATGT	
oATK5	*comK_Bco_*	GGTACCTCCGCATGCTGGAAGAAT	KpnI
oATK6	*comK_Bco_*	GGGCCCCAATTGCCCATGTTGCATAA	ApaI
oATK7	*comGA_Bco_*	GGTACCTTCCTGGACGGATACTTC	KpnI
oATK8	*comGA_Bco_*	GGGCCCTTCTACCGACATAATCCATC	ApaI
oATK13	*comK_Bco_*	GCAAAGCTTAGAGAGTGGATCATGAGATA	HindIII
oATK14	*comK_Bco_*	CAGGTCGACGGACTTGCAGTTCGCAATGT	SalI
oATK16	*rpsD_Bco_*	GGGTACCAATCCAGTAAACGGGACTTAT	KpnI
oATK17	*rpsD_Bco_*	GGGGCCCTTTCCAGCTTGGACCTGTAT	ApaI
oATK26	*comK_Bco_*	TGGGATCCATGGGGGAATGCATTATGCAA	BamHI
oATK48	*comC_Bco_*	ACGGGGCCCCGCAAAATAAGCTGTCCATA	KpnI
oATK49	*comC_Bco_*	ACGGGTACCATTTGCCGGAAATCGACGTG	ApaI
oATK50	*comFA_Bco_*	ACGGGGCCCCTGTTCGGAGAAAACAGAAG	KpnI
oATK51	*comFA_Bco_*	ACGGGTACCTGCCTGGATGCTGAAATAAG	ApaI
oATK83	*rpiA_Bco_*	AATAGCAGACTTGAACGACAC	
oATK84	*rpiA_Bco_*	CACCAAATGCTTGTATCCGA	
oATK87	*comGA_Bco_*	AAGCAGGCATTACTTATAGCAC	
oATK88	*comGA_Bco_*	GGACAACGCAATGTAATCAG	
oATK89	*comC_Bco_*	CCTCCTCTATCTCATTGCCT	
oATK90	*comC_Bco_*	GAAACGCAAATACATCCCGA	
comG1	*comGA_Bsu_*	CCGGAATTCATGGTGACCATGTCTGCT	
comG2	*comGA_Bsu_*	CGCGGATCCCTCTCCTTTCAACGC	
pK-F	*comK_Bsu_*	AATCTATCGACATATCCTGCAA	
KFPr	*comK_Bsu_*	GGAATTCTTGCGCCGTTCACTTCATAC	

### Construction of Promoter-*gfp* Reporter Plasmids

The *gfp* gene was first obtained from pSG1151 using KpnI-XbaI restriction enzymes and ligated into the corresponding sites of the broad host range pNW33N vector, resulting pATK2. pATK2 was digested with KpnI and ApaI and used to ligate the promoter fragments of *comK_Bco_* and *comGA_Bco_* obtained with PCR reaction using oligonucleotides oATK5 and oATK6 (for *comK_Bco_*) and oATK7 and oATK8 (for *comG_Bco_*) and digested with the same restriction enzyme pairs. The integrity of cloned fragments was verified by sequencing.

### Protein Overexpression and Purification

1 liter culture of cells containing the pMALcomK*_Bco_* or pMALcomK [53] was grown for 2 hours at 37°C and induced with 0.1 mmol l^−1^ of IPTG at 0.8 of OD_600_. Cells were harvested by centrifugation (10 min, 4°C, 6500×*g*). Pellets were washed with a buffer containing 1.17% NaCl, 25 mmol l^−1^ EDTA, 10 mmol l^−1^ NaN_3_, 0.15% DTT, 50 mmol l^−1^ Tris-HCl pH 7.4. Cells were lysed by sonification (15×10 s at 10 kHz with 30 s intervals), and the sonicated fractions were centrifuged (20 min, 4°C, 9000×*g*) to obtain a supernatant that contains the MBP-ComK. The fraction with the MBP-ComK has been loaded on an amylose column which had been equilibrated with a buffer containing 0.5 mM DTT, 20 mM Tris-HCl pH 8.0. Elution was performed with the same buffer, now containing 10 mM maltose. The fractions were stored immediately at 4°C (after analysis the fractions were pooled and stored at −80°C). The purity of MBP-ComK*_Bco_* and MBP-ComK*_Bsu_* was verified on SDS-PAGE and the purified proteins were also validated using Western hybridization with antibody raised against ComK*_Bsu_* as described in [53]. Sample preparation and Western hybridization on the *B. coagulans* samples were performed as described previously for *B. cereus* [11]. *B. coagulans* wild-type and *comK_Bco_* overexpression strains were grown in BC medium until 0.8 of OD_600_ and induced with 0.1 mmol l^−1^ IPTG. Three hours after induction, samples were harvested by centrifugation (10.397×*g*, 1 min, 4°C), disrupted using lysozyme treatment. The ComK*_Bco_* protein level was detected after SDS-PAGE using Western hybridization with ComK*_Bsu_*– specific antibody [53].

### Gel Retardation Assay

Gel retardation assays were carried out essentially as described by Susanna et al. [26]. The promoter regions of *B. coagulans* putative competence genes *comK*, *comG*, *comC* and *comFA* were obtained by PCR using oligos oATK5-oATK6, oATK7-oATK8, oATK48-oATK49, and oATK50-oATK51, respectively (Table 2). The *B. subtilis comK* and *comG* promoter fragments were obtained using oligos pK-F – KFPr [54] and comG1-comG2 [26], respectively. The *B. coagulans rpsD* promoter region was used as negative control. The resulting fragments were end-labeled with [γ-^33^P] ATP using T4 polynucleotide kinase (Roche Nederland B.V., The Netherlands). Purified MBP-ComK*_Bco_* and MBP-ComK*_Bsu_* proteins and probes were premixed on ice in binding buffer. Reaction mixtures contained poly (dI-dC) that is known to eliminate non-specific DNA binding of ComK [25]. Samples were incubated at 30°C, and were loaded on a 6% polyacrylamide gel after 20 min incubation. Gels were run in 1× TBE buffer (0,089 mmol l^−1^ Tris, 0,089 mmol l^−1^ Boric Acid, 0,022 mmol l^−1^ EDTA) at 90 V for 60 minutes, dried in a vacuum dryer and autoradiographed using phosphoscreens and a Cyclone PhosphorImager (Packard Instruments, Meridien, CT).

### Quantitative RT-PCR

*B. coagulans* wild type and *comK_Bco_* overexpression strains were grown in BC medium until 0.8 of OD_600_ and induced with 0.1 mmol l^−1^ IPTG. Two hours after induction, samples were harvested by centrifugation (10.397×g, 1 min, 4°C). A total of three independent biological replicates were included. RNA preparation of quantitative PCR was performed as described before [55], [56]. The pellets were immediately frozen in liquid nitrogen and stored at −80°C. RNA extraction was performed with the Macaloid/Roche protocol [57]. Samples were treated with RNase-free DNase I (Fermentas, St. Leon-Rot, Germany) for 60 min at 37°C in DNaseI buffer (10 mmol l^−1^ Tris·HCl (pH 7.5), 2.5 mmol l^−1^ MgCl_2_, 0.1 mmol l^−1^ CaCl_2_), and re-purified with the Roche RNA isolation Kit. RNA concentration and purity was assessed using NanoDrop ND-1000 Spectrophotometer (Thermo Fisher Scientific). Reverse transcription was performed with 50 pmol random nonamers on 4 µg of total RNA using RevertAid™ H Minus M-MuLV Reverse Transcriptase (Fermentas, St. Leon-Rot, Germany). Quantification of cDNA was performed on an CFX96 Real-Time PCR System (BioRad, Hercules, CA) using Maxima SYBR Green qPCR Master Mix (Fermentas, St. Leon-Rot, Germany). The following oligos were used: for *comGA*, oATK87 and oATK88, for *comC*, oATK89 and oATK90 and for *rpiA* gene of *B. coagulans*, oATK83 and oATK84 (oligo sequences are listed in Table 2). The amount of *comGA* and *comC* cDNA levels was normalized to the level of *rpiA* cDNA using the 2^−ΔΔCt^ method [58].

### Flow Cytometric Analyses and Microscopy

*B. subtilis* wild type and Δ*comK* strains carrying either pATK5 or pATK6 were grown ON in minimal medium supplemented with chloramphenicol (5 µg ml^−1^). For the flow cytometric analyses, cultures were inoculated into fresh minimal medium. Samples were taken after transition to stationer phase every hour. Cells were diluted 10 fold in minimal salts and analyzed on a Coulter Epics XL-MCL flow cytometer (Beckman Coulter Mijdrecht, NL) operating an argon laser at 488 nm. Green fluorescent protein (GFP) signals were collected through an FITC filter with the photomultiplier voltage set between 700 and 800 V. Date were obtained using EXPO32 software (Beckman Coulter) and further analyzed using WinMDI 2.8 (The Scripps Research Institute). Figures were prepared using WinMDI 2.8 and Adobe CS4 Illustrator.

The fluorescence of the GFP reporter protein was visualized with a Zeiss Axiophot microscope, using filter set 09 (excitation, 450 to 490; emission, >520 nm). Imaging of P*comGA_Bsu_*-*gfp* in individual cells using fluorescence microscopy was performed as described by Smits *et al.* [30] using AxioVs20 software (Zeiss) for image capturing and figures were prepared for publication using Adobe CS4 Illustrator.

### Nucleotide Sequence Accession Numbers

Sequences used in this study have been deposited in GenBank under accession numbers JX518619 (*comK_Bco_*), JX518620 (*comGA_Bco_*), JX518621 (*comC_Bco_*), JX518622 (*comFA_Bco_*), JX518623 (*rpsD_Bco_*), JX518624 (*rpiA_Bco_*).

## Supporting Information

Figure S1 **A. Schematic presentation of the promoter region of putative competence related genes.** Filled boxes indicate putative AT-boxes (maximum 3 mismatches to the consensus AAAA-N_5_-TTTT), open boxes indicate upstream open reading frames and *com* genes, numbers denote spacing between AT-boxes resulting in a so called K-box (8 bp and 31 bp in the case of *comK_Bco_* and *comC_Bco_*, respectively). **B. Sequences of** ***B. coagulans*** **DSM1 promoter regions related to competence.** Bold letters indicate putative AT-boxes. The putative open reading frames, *com* genes are indicated below the sequence.
(PDF)

Figure S2 **Detection of the ComK_Bco_ protein.** Equal amounts of proteins were loaded in each lane, Samples were taken from induced (+) or non-induced (−) cultures. Cells were centrifuged, lysed and analysed by Western blotting using ComK*_Bsu_*-specific antibodies. The arrows indicate ComK specific signal (K) and non-specific signal (NP). Positions of molecular weight marker bands are indicated on the right side of the gel.
(TIF)
