# Correction: Functional Analysis of the ComK Protein of *Bacillus coagulans*

**DOI:** 10.1371/annotation/16c64f49-b803-458d-8c8c-fa22a9c649b2

**Published:** 2013-02-14

**Authors:** Ákos T. Kovács, Tom H. Eckhardt, Mariska van Hartskamp, Richard van Kranenburg, Oscar P. Kuipers

Mariska van Hartskamp should have been listed as third author for this article, the authors therefore wish to revise the author list to read as follows:

Ákos T. Kovács, Tom H. Eckhardt, Mariska van Hartskamp, Richard van Kranenburg, Oscar P. Kuipers

Mariska van Hartskamp is affiliated at Purac, Gorinchem, The Netherlands.

The correct citation is: Kovács ÁT, Eckhardt TH, van Hartskamp M, van Kranenburg R, Kuipers OP (2013) Functional Analysis of the ComK Protein of Bacillus coagulans. PLoS ONE 8(1): e53471. doi:10.1371/journal.pone.0053471

The authors' contributions should be revised to read as follows in order to acknowledge Ms van Hartskamp's contribution: Performed the experiments: ATK, THE, MvH.

In addition, the affiliation details for Richard van Kranenburg should be revised to read as Laboratory of Microbiology, Wageningen University, Wageningen, The Netherlands and Purac, Gorinchem, The Netherlands. 

