# Supplementary material for: Functional Analysis of the ComK Protein of Bacillus coagulans
Source: PLoS One. 2013 Jan 3;8(1):e53471. doi: 10.1371/journal.pone.0053471 (PMC3536758; doi:10.1371/journal.pone.0053471)
Supplement: Figure S1 — A. Schematic presentation of the promoter region of putative competence related genes. Filled boxes indicate putative AT-boxes (maximum 3 mismatches to the consensus AAAA-N5-TTTT), open boxes indicate upstream open reading frames and com genes, numbers denote spacing between AT-boxes resulting in a so called K-box (8 bp and 31 bp in the case of comKBco and comCBco, respectively). B. Sequences of B. coagulans DSM1 promoter regions related to competence. Bold letters indicate putative AT-boxes. The putative open reading frames, com genes are indicated below the sequence. (PDF) [file pone.0053471.s001.pdf]

A

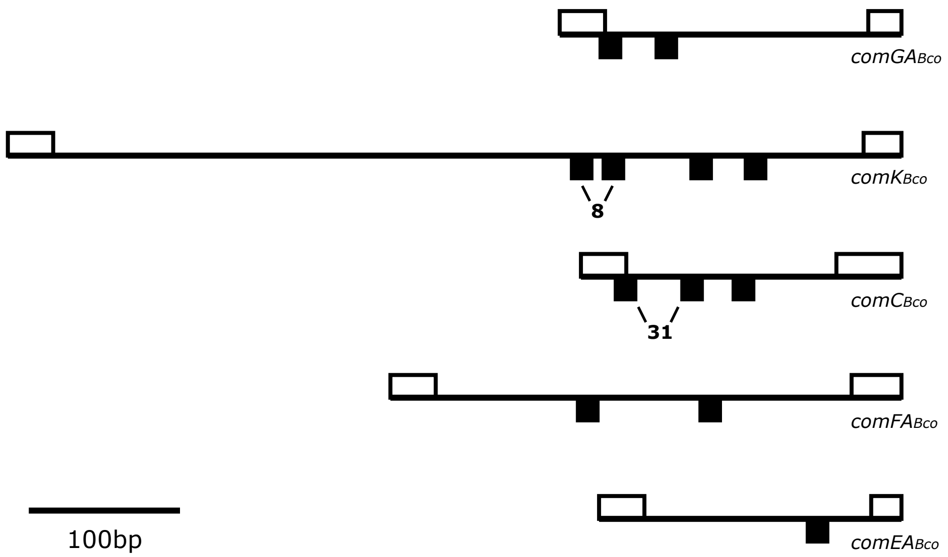

B

P*comGA*$_{Bco}$

1 aacaacgcca aaatttgtga caaaaat**cac acactttttt** gcggctgaga gaaaaaaata gcga**aaaaat ttttgctt**tc agcaggaatt ttgaaagccg

>>...........*orf*............>>

101 tgtcgaaatg agagaatatc ccaaaagaaa ggtggtatgt gatggattat gtcggtagaa aaaatcgcgg

>>.......*comGA*........>>

P*comK*$_{Bco}$

1 ctggagcgga tccgcatgct ggaagaataa ggaatactga aagtgcagca ggcgcggcct gttccgggca aaaatctttt ggccgtgccc tgcacctgcc

>>.............*yhfN*..........>>

101 atttttatgc aaaagaaaag ccatgcaaac gggccgggcc tgtttgcatg gcctgtattt atccaaattg caggttgatt tttttaagtt cgttcgttaa

201 atgcatgaac tgctcctcat cgcggagatc aagtacatgg tcaatttttc ttaataactc ttctcttttt ttgttcagca ggatctcatt cagcaacatg

301 tccgtgtaaa tgtgttccac aaatttctcc atctgaatcc gggacttcgg gctttctttc ggcacgtcag aga**aagattt tttgtt**ttcc atgt**atgatc**

401 **accttttt**tcg ctttttttta ttatacagag catttcaata aaaagcaatg ta**aaaattga atttt**tctga aacgattaac aaagaaaa**aa atgcatattt**

501 **t**taaaaaagt actagaaatg taggaagatt gtactataat agagagtgga tcatgagaaa aataaaatgg gggaatgcat tatgcaacat g

>>..........*comK*..........>>

P*comC*$_{Bco}$

1 agtgacccgg tcgcaataaa ggg**gcaataa gttttc**ggca tgttcatcct tgccattaca tatgctt**aat atcgcccgtt** gcttgccggt agcgaaccaa

>>...........*orf*............>>

101 g**aaaatacca tatg**ctgaaa gatcagaggg atcaaacatg catggattat ggacagctta ttttgcggca ctggggatgg

>>..................comC...................>>

P*comFA*$_{Bco}$

1 tgcctggatg ctgaaataag agccaggctg ttccaaatcg ctggaacagc ccggcaagat gggatcgtcc gggataaacc tggcataaca ggcccgggga

>>......*orf*.......>>

101 caagccttga aacctgccgg acag**ccaata ccttttg**agg attttagcga ttaagcgtat ccttcccggg acaagacctt aaccccgccg gccaatccgc

201 gattg**aataa gcagttgt**aa tccatgcatc cgtacaggct ttttcccgaa tccgtttgga accgggccgc cccatttgcc tacctccccc gcaattgaca

301 aaggagtgat cactttgcct tctgttttct ccgaacag

>>............*comFA*.............>>

P*comEA*$_{Bco}$

1 atttcccgtt ccgatcacgc caatgtccat gctgttttcc ctccccaagc cgccgatctt tcagcgcccg gtacaatata tgcgcagatc cgggcgatta

<<...........*comER*............<<

101 tgtatctgaa aaaagaaaaa cagaggtgaa ggtctttg**aa aacgttgttt c**tcaaatacc gctggcatgc tgctgcagct gtggcagcca ttacgattgc

>>......comEA......>>
