# Supplementary figures and images for: Functional Analysis of the ComK Protein of Bacillus coagulans
Source: PLoS One. 2013 Jan 3;8(1):e53471. doi: 10.1371/journal.pone.0053471 (PMC3536758; doi:10.1371/journal.pone.0053471)

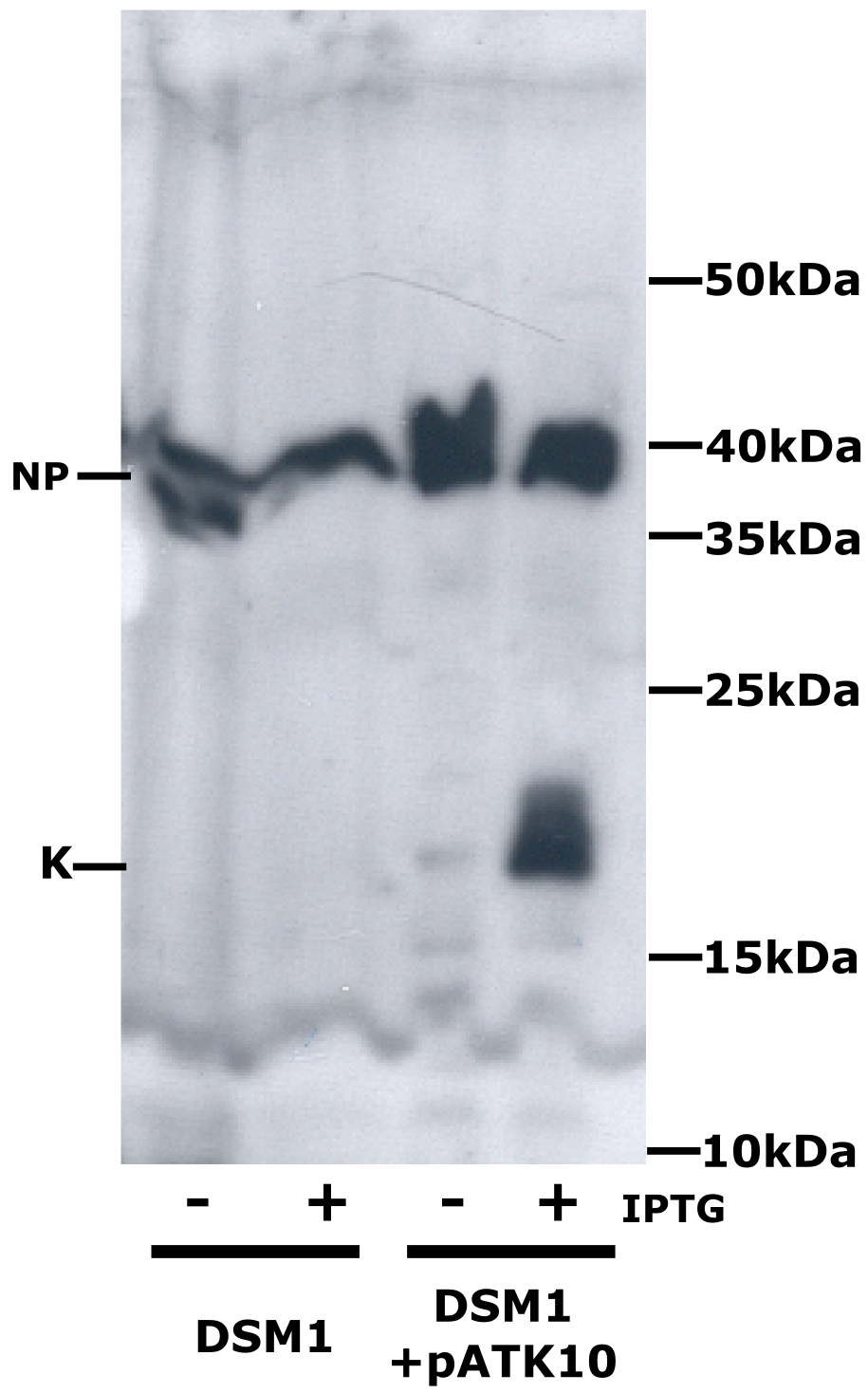

Supplement: Figure S2 — Detection of the ComKBco protein. Equal amounts of proteins were loaded in each lane, Samples were taken from induced (+) or non-induced (−) cultures. Cells were centrifuged, lysed and analysed by Western blotting using ComKBsu-specific antibodies. The arrows indicate ComK specific signal (K) and non-specific signal (NP). Positions of molecular weight marker bands are indicated on the right side of the gel. (TIF) [file pone.0053471.s002.tif]
